# Development of Interspecific Hybrids between a Cultivated Eggplant Resistant to Bacterial Wilt (*Ralstonia solanacearum*) and Eggplant Wild Relatives for the Development of Rootstocks

**DOI:** 10.3390/plants9101405

**Published:** 2020-10-21

**Authors:** Mohamed Rakha, Ahmed Namisy, Jaw-Rong Chen, Mohammed E. El-Mahrouk, Elmahdy Metwally, Naglaa Taha, Jaime Prohens, Mariola Plazas, Dalia Taher

**Affiliations:** 1 Horticulture Department, Faculty of Agriculture, University of Kafrelsheikh, Kafr El-Sheikh 33516, Egypt; d108035002@mail.nchu.edu.tw (A.N.); mohamed.elmahrouk@agr.kfs.edu.eg (M.E.E.-M.); elmahdy.metwali@agr.kfs.edu.eg (E.M.); 2 World Vegetable Center, P.O. Box 42, Tainan 74199, Taiwan; jaw-rong.chen@worldveg.org; 3 Department of Plant Pathology, National Chung Hsing University, Taichung 40277, Taiwan; 4 Research Institute of Plant Pathology, Agricultural Research Centre (ARC), Giza 12619, Egypt; yousry.omeer@agr.kfs.edu.eg; 5 Joint Research Unit for the Valorization and Breeding of Horticultural Landraces, Instituto de Conservación y Mejora de la Agrodiversidad Valenciana, Universitat Politècnica de València, 46022 Valencia, Spain; 6 Joint Research Unit for the Valorization and Breeding of Horticultural Landraces, Fundació Miquel Agustí, BarcelonaTech, 08860 Castelldefels, Spain; maplaav@btc.upv.es; 7 Vegetable Crops Research Department, Horticulture Research Institute, Agriculture Research Center, Giza 12619, Egypt; daliataher1981@gmail.com

**Keywords:** biotic stress, disease resistance, embryo rescue, *Solanum melongena*, SSR markers, wild accessions

## Abstract

Bacterial wilt caused by *Ralstonia solanacerum* is one of the most economically and destructive eggplant diseases in many tropical and subtropical areas of the world. The objectives of this study were to develop interspecific hybrids, as potential rootstocks, between the eggplant (*Solanum melongena*) bacterial wilt resistant line EG203 and four wild accessions (*S. incanum* UPV1, *S. insanum* UPV2, *S.* *anguivi* UPV3, and *S. sisymbriifolium* UPV4), and to evaluate interspecific hybrids along with parents for resistance to bacterial wilt strains Pss97 and Pss2016. EG203 was crossed successfully with wild accessions UPV2 and UPV3 and produced viable seeds that germinated when wild accessions were used as a maternal parent in the crosses. In addition, viable interspecific hybrids between EG203 and UPV1 were obtained in both directions of the hybridization, although embryo rescue had to be used. Hybridity was confirmed in the four developed interspecific hybrid combinations with three SSR markers. EG203 was resistant to both strains Pss97 and Pss2016, while UPV1 and UPV3 were, respectively, resistant and moderately resistant to Pss2016. The four interspecific hybrids with UPV2, UPV3, and UPV1 were susceptible to both bacterial wilt strains, indicating that the resistance of EG203, UPV1, and UPV3 behaves as recessive in interspecific crosses. However, given the vigor of interspecific hybrids between eggplant and the three cultivated wild species, these hybrids may be of interest as rootstocks. However, the development of interspecific hybrid rootstocks resistant to bacterial wilt will probably require the identification of new sources of dominant resistance to this pathogen in the eggplant wild relatives.

## 1. Introduction

Eggplant (*Solanum melongena* L.) is one of the most important vegetables in the world, ranking as the sixth vegetable crop in production, after tomato (*Solanum lycopersicum* L.), watermelon (*Citrullus lanatus* (Thumb.) Matsum. & Nakai), onion (*Allium cepa* L.), cabbage (*Brassica oleracea* L.), and cucumber (*Cucumis sativus* L.), with a global production 54.07 million tonnes [1]. Eggplant is one of the vegetables with the highest antioxidant capacity, due to the high contents in fruit phenolics and flavonoids [2,3].

Bacterial wilt caused by *Ralstonia solanacerum* is one of the most important eggplant diseases in tropical and subtropical regions in the world. The occurrence of the bacterial wilt disease is known in tropical and subtropical regions of the world [4]. It grows well from 28 °C to 32 °C strictly in aerobic conditions [5], and is able to infect eggplant and other important Solanaceae crops, such as potatoes, tomatoes, and peppers [6]. The pathogen strains are divided into four phylotypes [7], which are genetically different corresponding to the geographical area of origin: Phylotype 1 strains originate from Asia, phylotype 2 from the Americas, phylotype 3 from Africa and surrounding islands, and phylotype 4 from Indonesia.

Bacterial wilt is a very serious soil-borne disease, due to its wide geographical distribution and unusually broad host range of more than 200 plant species belonging to 53 different families [7]. In addition, *R. solanacearum* can survive in the soil for many years [8]. The pathogen enters the plant through wounds in the roots caused by transplanting, nematodes, insects, and through natural wounds. Thereafter it starts multiplication rapidly in the vascular system; finally, the xylem elements are filled with bacterial cells, infected plants suffer yellowing, wilting, and often die rapidly [9].

Bacterial wilt is very difficult to control. However, several managing strategies, including soil disinfection, soil amendment, biological and chemical controls, resistant cultivars, or rootstocks for grafting, have been tested for the control of bacterial wilt [10,11,12]. Chemical control is not economically practical for treating bacterial wilt, especially in the field, due to the localization of pathogen inside xylem and its ability to survive deep in the soil [13,14]. A study tested bactericides under both greenhouse and open field conditions [14], and it was found that antibiotics, such as penicillin, ampicillin, tetracycline, and streptomycin had little effect on the pathogen. Another study on biological control [15] reported that different strains of *Pseudomonas fluorescens, Bacillus subtilis, B. amyloliquefaciens*, and rhizobacteria could contribute to reducing the incidence of soil-borne diseases, including bacterial wilt.

Breeding for resistance to bacterial wilt is still the most appropriate, economical, and environmentally promising strategy for controlling this pathogen [9,16]. Host resistance to *R. solanacearum* has been studied in eggplant since the 1960s in eggplant [17,18,19], and molecular markers linked to the resistance of several sources have been developed [20,21]. A recent study [22] detected several QTLs associated with resistance to phylotypes I and III in the cross between the resistant EG203 accessions and a susceptible eggplant, although QTLs expression was highly influenced by environmental conditions. Wild relatives have been frequently used in the genetic improvement of many crops, as sources for resistance to biotic and abiotic stresses and also for quality traits [23,24], as well as for the development of rootstocks.

The objectives of the current study were to develop interspecific hybrid rootstocks between cultivated eggplants and wild relatives (*S. incanum* L., *S. insanum* L., *S. anguivi* Lam., and *S. sisymbriifolium* Lam.), and to evaluate the interspecific hybrids along with parents for resistance to the highly aggressive isolates of *R. solanacearum* Pss97 and Pss2016 for breeding and development of resistant rootstocks that can be used globally to manage bacterial wilt disease sustainably.

## 2. Results and discussion

### 2.1. Production of Interspecific Hybrids

**Figure 1:**
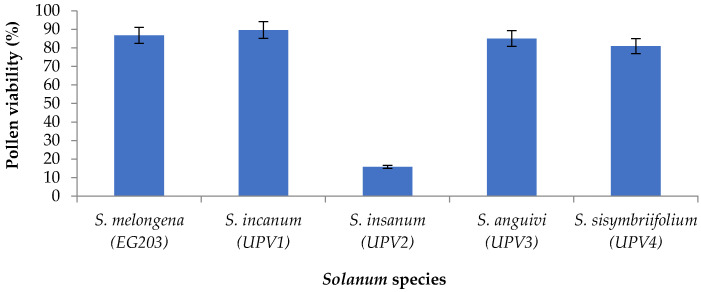
Pollen viability (%; mean ± SE) in cultivated eggplant (*Solanum melongena* accession EG203) and wild accessions (*Solanum incanum* UPV1, *Solanum insanum* UPV2, *Solanum anguivi* UPV3, and *Solanum sisymbriifolium* UPV4) used in interspecific hybridizations.

The results of pollen viability in cultivated eggplant and wild accession are presented in Figure 1. Cultivated eggplant EG203 had high pollen viability (86.76%). Pollen viability in wild accessions was variable depending on the species. The highest pollen fertility was observed in *S. incanum* UPV1 (89.65%), followed by *S. anguivi* UPV3 (85.03%) and *S. sisymbriifolium* UPV4 (80.94%). On the contrary, *S. insanum* UPV2 had very low pollen fertility (15.78%).

The results of the interspecific hybridization between the cultivated eggplant EG203 and wild species with respect to mean fruit set (%), number of seeds per fruit, and seed germination (%) are presented in Table 1. Cultivated eggplant EG203 was successfully crossed with wild accessions when used as the male parent, except in crosses with *S. sisymbriifolium* UPV4. However, when *S. melongena* EG203 was used as the female parent, crosses were successful only with *S. incanum* UPV1. The percentage of fruit set of interspecific hybridizations was variable depending on the direction of the hybridization and the wild species involved. The maximum fruit set (68.8%) was recorded in the cross *S. incanum* UPV1 × *S. melongena* EG203 followed by *S. melongena* EG203 × *S. incanum* UPV1 (65.2%), *S. anguivi* UPV3 × *S. melongena* EG203 (46.2%) and *S. insanum* UPV2 × *S. melongena* EG203 (40.0%). When using EG203 as a male parent, the largest amount of seeds per fruit was obtained in hybridizations with *S. insanum* UPV2, with an average value of 0.85 g/fruit, which is equivalent to more than 250 seeds/fruit, followed by hybridizations with *S. anguivi* UPV3 (0.30 g/fruit), which is equivalent to around 70 seeds/fruit. Seed quantity per fruit in the hybridization with *S. incanum* UPV1 was 0.03 g/fruit, which is equivalent to 17 seeds/fruit. When using *S. melongena* EG203 as a female parent, a large number of seeds were obtained with the only wild accession (*S. incanum* UPV1) in which the crosses were successful.

Considering the three wild species (*S. incanum*, *S. insanum*, and *S. anguivi*) for which we were able to obtain interspecific hybrids with *S. melongena*, in general, our results are in agreement with previous studies [25]. In this respect, Devi et al. [26], found that *S. incanum* was highly crossable with the cultivated eggplant genotypes. In another study, Plazas et al. [27] also reported that crosses between *S. melongena* and *S. incanum*, as well as with *S. insanum* and *S. anguivi*, were successful in both directions, and the highest rate of fruit set was obtained when using *S. melongena* as the female parent with *S. insanum*, which presented a fruit set above 15%. When using *S. melongena* as the male parent, the highest fruit set was obtained with *S. anguivi* with values above 25%. In another study, reciprocal crosses were also successful between *S. melongena* and *S. incanum* [28]. However, in contrast to our results and those of Plazas et al. [27], Afful et al. [29] reported that no fruit set was observed when several *S. melongena* cultivars were crossed as the female parent with *S. anguivi*. The variation between our results and those of previous studies may be related to the different genetic background of the parental lines [30], to irregular chromosome associations during the formation of gametes [31,32], or to different environmental conditions.

The crosses we performed between *S. melongena* and *S. sisymbriifolium* were not successful in any of the directions (Table 1). In another work, Plazas et al. [27] found that a low fruit set was obtained after crossing *S. melongena* and *S. sisymbriifolium*; however, these fruits were parthenocarpic. This result confirms that *S. sisymbriifolium* is very distant from eggplant [33], which is considered a tertiary gene pool species. However, hybrids and backcrosses of *S. melongena* with another tertiary gene pool species (*S. elaeagnifolium* Cav.) have been obtained by García-Fortea et al. [34] through embryo rescue.

We found that seed germination percentage among the interspecific hybrids was very high for those involving *S. melongena* as the male parent, and *S. insanum* UPV2 (90%), as well as *S. anguivi* UPV3 (88%) as females. However, no germination was observed in the cross between *S. melongena* EG2013 with *S. incanum* UPV1 in both directions (Table 1). Furthermore, most of the seeds in these two interspecific hybrids looked as if they were incompletely developed or immature. In a study in which crosses were made between *S. incanum* and *S. melongena*, Anis et al. [35], found that unilateral hybrids could be obtained when *S. incanum* was used as the female parent. In contrast, Plazas et al. [27] found that a high seed germination was obtained for the hybrids between *S. melongena* and *S. incanum*. In addition, Devi et al. [26] reported that interspecific hybrids of *S. melongena* with *S. incanum* produced viable and highly vigorous plants with earliness and higher yield. These differences in germination among different studies could be explained by the different genetic background of parental lines despite the same species combination. Moreover, in our case, the abnormal seeds (empty seeds, seeds with a different appearance, low weight) obtained from the interspecific crosses might also have contributed to the lack of germination observed. In this way, in other species, seeds have been reported to contain an under-developed embryo and/or endosperm, which prevent germination [36].

**Figure 2:**
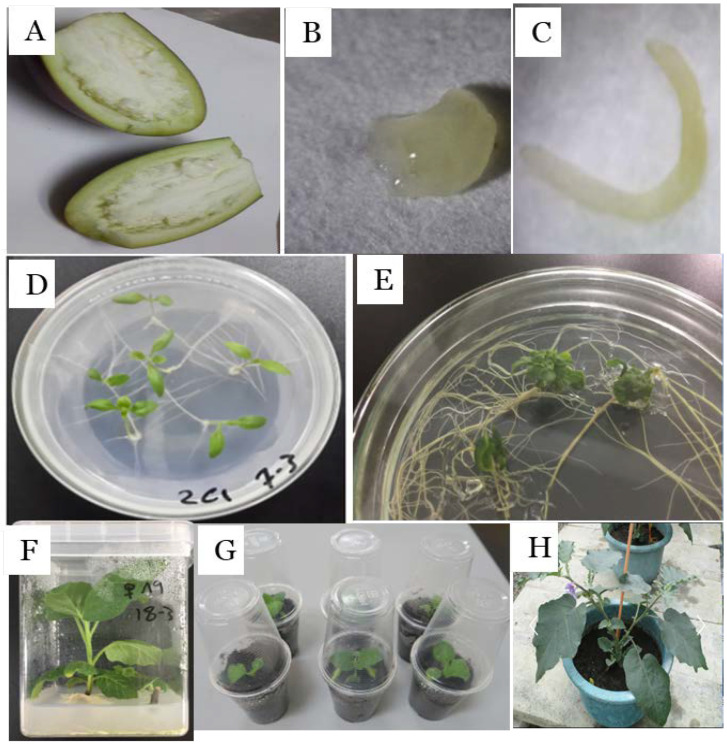
Embryo rescue technique used: (**A**) immature fruit come from crosses between *S. melongena* and *S. incanum*; (**B**); heart embryo stage; (**C**) torpedo embryo stage; (**D**) plantlets germinated directly from an embryo; (**E**) abnormal plantlets; (**F**) normal plant grown in a jar; (**G**) acclimatized plants; and (**H**) mature acclimatized hybrid plant grown in a greenhouse. The diameter of the Petri dishes in **D** and **E** is 9 cm. The flask in **F** has a diameter of 6.5 cm and a height of 9.8 cm. The glasses in **G** have a mouth diameter of 5.8 cm and a capacity of 100 cm^3^. The pot in H has a mouth diameter of 30 cm and a capacity of 5 L.

Given that no viable seeds were obtained in our hybridizations between *S. melongena* EG203 and *S. incanum* UPV1 in both directions, embryo rescue was employed to obtain hybrids from the crosses between cultivated eggplant EG203 and UPV1 (Figure 2). In this respect, embryo rescue has been widely used for producing plants from hybridizations in which failure of the endosperm to develop properly causes embryo abortion [37]. The responses obtained by us of three different tissue culture media (M1–M3) for germinating embryos from the crosses between *S. melongena* EG203 and *S. incanum* UPV1 at three different stages (globular, heart, and torpedo stage) are presented in Table 2. The results showed that the torpedo stage was the best embryo stage to germinate interspecific hybrid plantlets, with regeneration efficiency from 78.9% to 88.5% when *S. melongena* EG203 is used as the female parent and 20.5% to 32.6% when *S. incanum* UPV1 is used as the female parent. In contrast, the globular stage in both directions of interspecific hybridizations did not produce any plantlets under any of the three media used in this study. Regarding culture media, the MS medium supplemented with 0.01 IAA and 0.1 Kin (MS2) was the best medium to develop plantlets from embryos, with regeneration efficiency reaching up to 88.5% when EG203 was used as the female parent and 32.6% when UPV1 was used as the female parent. However, several abnormal plantlets were observed in this same MS2 medium. The MS medium without growth regulators (MS1) produced the lowest number of normal plantlets. Other works have evaluated the use of embryo rescue and different media for obtaining interspecific hybrids of eggplant, obtaining a diversity of results. In this respect, Verba et al. [38] found that embryos of the combination *S. melongena × S. integrifolium* developed into normal plantlets when torpedo embryos were cultured on MS medium with TDZ (0.1 mg/L). In another work, Sharma et al. [39] achieved partial success in obtaining hybrid plants between *S. melongena* × *S. sisymbriifolium* when torpedo embryos were cultured on Nitsch and Nitsch [40] medium; however, the plants could not survive for a very long time and died. Conversely, in the interspecific crosses between *S. melongena* and *S. khasianum* Clark [41], fertile hybrids were obtained when embryos 25 days old were cultured on Nitsch and Nitsch medium. Embryo rescue by culturing immature ovules 15 to 27 days old after pollination on MS medium without growth regulators was also used by Bletsos et al. [42] to obtain hybrids between *S. torvum* Sw. and *S. melongena*. These results indicate that the success and efficiency of embryo rescue in interspecific hybrids of eggplant largely depend on the specific hybrid combination and media used.

### 2.2. Hybridity Confirmation

We genotyped the four interspecific cross combinations obtained and their parents with three SSR markers to confirm hybridity. The number of alleles scored was three for smSSR1, and two for both EPSSR04 and EPSSR133 (Table 3). For SSR marker smSSR01 alleles, 300 bp and 290 bp were specific, respectively, of *S. melongena* EG203 and *S. anguivi* UPV3, while *S. incanum* UPV1 and *S. insanum* UPV2 shared the same allele (270 bp). For SSR marker EPSSR04, two profiles were observed among the four parents; one allele (320 bp) was shared between *S. melongena* EG203 and *S. incanum* UPV1, while the other (300 bp) was shared between *S. insanum* UPV2 and *S. anguivi* UPV3. Finally, two profiles were also observed for marker EPSSR133. In this case, one allele (240 bp) was specific to *S. insanum* UPV2, while the three other accessions had the other allele (220 bp). Considering the three SSR markers, each of the four parents had a unique profile, and *S. melongena* EG203 could be distinguished from the three wild parents with one (*S. incanum* UPV1), two (*S. anguivi* UPV3), or three (*S. insanum* UPV2) markers. The hybrids displayed the expected genotype, being heterozygote for the markers that were polymorphic among the two parents. As expected with nuclear SSRs, the genotyping results of the reciprocal crosses between *S. melongena* EG203 and *S. incanum* UPV1 were the same. The results indicate that, as found in other works [27], a limited number of SSR markers are sufficient for confirming hybridity in interspecific crosses of eggplant wild relatives.

### 2.3. Assessment of Disease Resistance

The resistance reaction and category of the susceptible control, four parents, and interspecific hybrids to *R. solanacearum* strains Pss97 and Pss2016 at four weeks after inoculation is presented in Table 4. The susceptible check (EG048) displayed the expected reactions to strains Pss97 and Pss2016, and all EG048 plants wilted and died rapidly at two and three weeks after inoculation by Pss97 and Pss2016, respectively. EG203 parent was resistant to both strains with 11.1% of wilting percentage (W%) and 9.9% of disease index (DI%) for the Pss97 strain, and 33.3% W%, and 20% of DI% for the Pss2016 strain. *Solanum incanum* UPV1 displayed a resistant reaction against Pss2016, while *S. anguivi* UPV3 was moderately resistant against this same strain. However, both of them were susceptible against strain Pss97. *Solanum insanum* UPV2 was susceptible against both strains. However, all interspecific hybrids were susceptible to both strains, with W% and DI%, ranging between 56.4% and 100%. The symptoms appeared one week after inoculation, and most of the plants were completely wilting after three weeks. These results indicate that the resistances in *S. melongena* EG203 and *S. incanum* UPV1 and the moderate resistance of *S. anguivi* UPV3 against strain Pss2016 must be controlled by a recessive gene(s). However, Salgon et al. [22] found that in an intraspecific cross, the resistance of EG203 was dominant, although strains of *R. solanacearum* different to ours were used. This suggests that the mode of inheritance of the resistance of EG203 to bacterial wilt depends, either on the strain, the genetic background, or both. Given our results, we suggest the evaluation of further germplasm accessions in *S. incanum* and *S. anguivi* to detect new sources of dominant resistance to bacterial wilt. In this respect, Ano et al. [44] demonstrated that high levels of resistance can be introgressed from one source of resistance of one related species *S. aethiopicum* L., whose wild ancestor is *S. anguivi*, into the genetic background of *S. melongena*. In any case, despite the susceptibility to bacterial wilt of the four interspecific hybrids obtained, given the vigor of interspecific hybrids of *S. melongena* with *S. incanum*, *S. insanum*, and *S. anguivi* [45], they might be of interest to be used as rootstocks in areas free from bacterial wilt.

## 3. Material and methods

### 3.1. Production of Interspecific Hybrids

Materials included for interspecific hybridization included bacterial wilt resistant line *S. melongena* EG203 and four wild accessions, namely, *S. incanum* UPV1, *S. insanum* UPV2, *S. anguivi* UPV3, and *S. sisymbriifolium* UPV4 (Table 5) were performed. For the pollen viability test, the mature flower buds were covered with a paper bag a day before anthesis. On the day of anthesis, flowers were collected at 8 a.m. Pollen viability was tested with acetocarmine (0.5%). Pollen samples were stained for 10 min and then observed under microscope (40× magnification). Viable pollens were stained and showed coloration, while non-viable pollen remained colorless. Both cultivated eggplant and wild accessions were used as the female and male parents in reciprocal crosses to develop interspecific hybrids. Over 400 crosses were performed. Crosses were made from 7 July to 27 October 2017 at the greenhouse with a maximum average temperature of 35.5 °C and a humidity of 84%. The female buds were emasculated a day before anthesis. The anthers were removed by forceps, and the buds were covered immediately with a paper bag to avoid the contamination of foreign pollen. Emasculations and hybridizations were made in the morning (07:00 to 09:00 a.m.). Pollen of the male parent was collected on a glass slide and gently applied by rubbing over the stigma of the emasculated flowers, which were covered again after pollination with the same bag paper and tagged. Pollinations of emasculating flowers were done twice for every flower in two successive days. The fruits obtained by the crosses were harvested at physiological maturity. Seeds were extracted manually in the laboratory from each fruit, and were dried in a drying room with temperature 15 ± 2 °C and humidity 15%. Data of fruit set (%), number of seeds/fruit, the weight of seeds/fruit, and germination (%) of F_1_ seeds were recorded.

Since seeds obtained from the reciprocal crosses between *S. melongena* and *S. incanum* UPV1 did not germinate, embryo rescue culture was applied to develop interspecific hybrids from this cross. Crosses were performed as indicated above. Fruits were harvested 15–35 days after pollination and brought to the laboratory. Fruits were washed and surface sterilized by ethanol (70%) under laminar flow cabinet conditions. Then, immature seeds were taken and deposited on sterile paper on the platform for the binocular magnifying glass. Embryos were excised carefully with hypodermic sterilized needles and immediately cultured in 90 × 15 mm petri dishes containing MS medium [43], supplemented with 40 g/L sucrose without or with growth regulators as follow; M1 (free MS medium), M2 (indole-3-acetic acid (IAA) at 0.01 mg/L) and Kinetin (Kin) at 0.1 mg/L Kin, and M3 (IAA at 0.01 mg/L and GA3 at 0.01 mg/L). MS media was obtained from Duchefa Biochemie (The Netherlands), and pH adjusted by NaOH and HCl (0.1 N) to 5.7 ± 0.1, and solidified with gelrite (2 g/L) before autoclaving at 121 °C under 1.1 kg/cm^2^ for 20 min. Petri dishes contained 20 mL medium, and five embryos were placed per Petri dish. The dishes were sealed with parafilm and incubated in a growth chamber under constant temperature (25 ± 1 °C). Illumination was provided by LED lamps with a blue light range of wavelengths 440 to 460 nm, dark red light with wavelengths range 650 to 670 nm, and light intensity 2601 lux for 16 h/day. After four weeks, hybrid plantlets were transferred to jars containing 25 mL free MS medium for four weeks. The efficiency of media and genotypes was evaluated in terms of the percentage of embryos that evolved to normal plantlets (by showing an early development of root and shoot) and abnormal plantlets (abnormally cotyledons, without the stem and with a powerful root system or without any roots). The abnormal plantlets or developed from callus were considered as not germinated. Plant adaptation proceeded according to the following order: (1) After four weeks, when plantlets became strong and had long and rolled root systems, the root system was cut off, and shoots were transplanted into a plastic container containing half-strength MS medium and kept in the light intensity of 2601 lux for 16 h until establishing good root system (2 or 3 roots at 1.2 mm length); (2) the plants were washed under tap water and cultured in pots containing autoclaved sterilized peat moss and covered with a clear plastic cup to maintain its relative humidity; and (3) finally they were placed in the growth chamber for one week under constant temperature (25 ± 1 °C) and light intensity of 2601 lux with a photoperiod of 16/8 h (light/dark). After that, the plants were transferred to the greenhouse.

### 3.2. Hybridity Confirmation

Three Simple Sequence Repeat (SSR) molecular markers (Table 6) were used to confirm hybridity in plants derived from the interspecific crosses. DNA of parental accessions and hybrids was extracted from fresh young leaves, as described by Fulton et al. [46]. The PCR reaction volume for a total of 10 μL consisted of 100 ng of genomic DNA as the template, 0.25 μM of each forward and reverse primers, 10× Supertherm GOLD buffer with 15 mM MgCl_2_ (JMR-470; Bersing, Technology, Zhunan Township, Taiwan), 0.125 units of *Taq* DNA polymerase (JMR-851; Bersing Technology, Zhunan Township, Taiwan), 200 μM of each dNTPs (P-2.5M; Focus Bioscience, Brisbane, QLD, Australia). The conditions for PCR were as follows: An initial denaturing step was performed at 94 °C for 10 min followed by 35 cycles at 94 °C for 30 s, a 45 s annealing at 50 or 55 °C, an extension at 72 °C for 45 min and a final extension at 72 °C for 5 min. All reactions were performed on an Eppendorf Mastercycler ep384 (Eppendorf, Hamburg, Germany). PCR products (10 µL) were run on a 6% acrylamide gels, visualized by ethidium bromide at 1 μg/mL, and photographed using a Cambridge Gel Documentation System documentation unit (Uvitec, Cambridge, UK) under UV light.

### 3.3. Assessment of Disease Resistance

Four interspecific hybrids (EG203 × UPV1, UPV1 × EG203, UPV2 × EG203, and UPV3 × EG203) along with their parents were evaluated for resistance to two strains of bacterial wilt (Pss97 and Pss2016) as described in Namisy et al. [47]. Eggplant accession EG048 was used as a susceptible check. Seeds or plantlets from in vitro culture were sown or transplanted, respectively, in 9-inch diameter plastic pots containing steam-sterilized soil mixture (3:1:1:1 ratio of soil, rice hulls, sand, and compost) and moved to the greenhouse with a photoperiod of 16/8 h day/night, the maximum average temperature of 34.8 °C and humidity of 86.9%, for evaluation. Seedlings were watered daily and fertilized weekly with an NPK fertilizer. Plants were arranged randomly, according a randomized complete block design (RCBD) with three replications. Due to variations in seed germination, 9 to 24 plants per accession or interspecific hybrid were tested against bacterial wilt strain. Four-week-old plants (4–6 fully expanded true leaves) were tested for *R. solanacearum* resistance.

Bacterial wilt strains Pss97 and Pss2016 were collected from eggplant and eggplant rootstock in Pingtung and Yilan Counties, respectively, in Taiwan [47], and both strains belong to the predominant virulence group, but Pss2016 has higher virulence than Pss97. These strains have been identified as genotype race 1, biovar 3, phylotype 1 based on identification conducted through host range [48], biovar test [49,50], and molecular markers [6], at the Bacteriology unit of WorldVeg. Bacterial strains were stored at −80 °C and cultured on 2,3,5-triphenyl tetrazolium chloride-amended medium TTC [51], and incubated at 30 °C for 2 days. Then several typical fluid white colonies with the pink center were transferred from TTC to medium 523 [52,53], and incubated at 30 °C overnight for multiplication. Bacterial mass from overnight cultures was transferred and suspend in water, adjusted the concentration until the optical density (OD) value reach 0.3 at the wavelength of 600 nm (about 10^8^ cfu/mL).

Before inoculation, roots of accessions and checks were injured with a knife by cutting through the soil 1 to 2 cm away from the stem base. A mount of 40 mL of bacterial suspension (10^8^ cfu/mL) was poured into each pot and kept the inoculated plants in a plastic greenhouse [54]. Plants were evaluated once a week for four weeks using the wilting percentage (W%) and disease index (DI) based on a disease rating scale (0–5), where 0 = no symptoms, 1 = one leaf partially wilted, 2 = two or three leaves wilted, 3 = all leaves wilted except the top two or three leaves, 4 = all leaves wilted, 5 = plant dead [55]. Wilting percentage (W%) was calculated following the formula:

(1) $$W\%\;=\;(\frac{Nw}{Nt})\text{ × }100$$

where Nw is the number of wilted plants (the plants with rating scale 1–5); and Nt is the total number of plants. The disease index (DI) was calculated using the following formula:

(2) DI = [(N0×0 + N1×1 + N2×2 + N3×3 + N4×4 + N5×5)Nt5] × 100

where N0 to N5 is the number of plants having disease rating scale values from 0 to 5; and Nt is the total number of plants. Accessions with DI from 0% to 30% were considered as resistant (R), above 30% to 40% as moderately resistant (MR), above 40% to 50% as moderately susceptible (MS), and over 50% as susceptible (S).

## 4. Conclusions

We have found that the bacterial wilt resistant EG203 variety of eggplant can be successfully hybridized with some wild species of interest for eggplant breeding from the primary and secondary gene pools. Although *S. incanum* UPV1 and *S. anguivi* UPV3, displayed resistance and moderate resistance, respectively, to one of the strains (Pss2016), the hybrids were susceptible to bacterial wilt, indicating that the resistance of EG203, UPV1, and UPV3 is recessive. Therefore, interspecific hybrids between these sources of resistance may have little value as rootstocks in areas affected by bacterial wilt. However, interspecific rootstocks are among the most popular rootstocks for improving plant vigor and yield of grafted scion in commercial production of solanaceous and cucurbitaceous crops [56,57,58,59,60]. In particular, the interspecific hybrids between *S. melongena* and the three wild species used here have proved to be heterotic for vigor traits [44] and might be of interest for areas free from bacterial wilt, although tests of graft affinity should be performed to confirm the potential interest of these hybrids [61]. The development of segregant generations from these hybrids may shed light on the genetic control of resistance and tolerance bacterial wilt and may pyramid the resistance from EG203 and the tolerance from the wild species. However, further studies are needed to confirm if durable resistance to bacterial wilt can be obtained through this strategy. Complementarily, the search for new sources of resistance for resistance to bacterial wilt expressed as dominant in interspecific hybrids could contribute to the development of new improved and resistant rootstocks.

## Figures and Tables

**Table 1:** Fruit set, seeds per fruit, and germination in interspecific hybridizations between *Solanum melongena* EG203 and wild eggplant relatives.

Wild Relative Parents	Number of Crosses	Fruit Set (%)	Seeds/Fruit Mean (g)	Germination (%) ^a^
*S. melongena* (male parent)				
*S. incanum* UPV1	32	68.8	0.03	0
*S. insanum* UPV2	40	40.0	0.85	90
*S. anguivi* UPV3	52	46.2	0.30	88
*S. sisymbrifolium* UPV4	66	0.00	ND	ND
*S. melongena* (female parent)				
*S. incanum* UPV1	46	65.2	1.58	0
*S. insanum* UPV2	40	0.0	ND	ND
*S. anguivi* UPV3	50	0.0	ND	ND
*S. sisymbrifolium* UPV4	52	0.0	ND	ND

**^a^** ND indicates no data.

**Table 2:** Effect of tissue culture media on germination (%) of different embryo stages and abnormal plants of the reciprocal hybrids between *S. melongena* EG203 and *S. incanum* UPV1. The number of embryos used for each embryo stage and medium is indicated (N).

Medium ^a^	Globular Stage		Heart Stage		Torpedo Stage		Abnormal Plants	
	N	%	N	%	N	%	N	%
*S. melongena* EG203 × *S. incanum* UPV1								
M1	30	0.0	25	32.0	15	86.7	0	0.0
M2	30	0.0	30	43.3	35	88.6	1	0.2
M3	30	0.0	30	56.7	38	78.9	0	0.0
*S. incanum* UPV1 × *S. melongena* EG203								
M1	30	0.0	24	8.3	39	20.5	0	0.0
M2	30	0.0	24	29.2	49	32.7	3	13.0
M3	30	0.0	24	16.7	52	30.8	0	0.0

**^a^** M1 (MS [43] free of growth regulators), M2 (MS supplemented with 0.01 mg/L IAA and 0.1 mg/L Kin) and M3 (MS supplemented with 0.01 mg/L IAA and 0.01 mg/L GA_3_).

**Table 3:** Polymorphic bands of Simple Sequence Repeat (SSR) markers in parents and their interspecific hybrids for hybridity confirmation.

Genotype	Allele Size		
	smSSR01	EPSSR04	EPSSR133
*S. melongena* EG203	300	320	220
*S. incanum* UPV1	270	320	220
*S. insanum* UPV2	270	300	240
*S. anguivi* UPV3	290	300	220
*S. melongena* EG203 × *S. incanum* UPV1	270, 300	320	220
*S. incanum* UPV1 × *S. melongena* EG203	270, 300	320	220
*S. melongena* EG203 × *S. insanum* UPV2	270, 300	300, 320	220, 240
*S. melongena* EG203 × *S. anguivi* UPV3	290, 300	300, 320	220

**Table 4:** Evaluation of a susceptible control of *S. melongena* (EG048), the parents of the interspecific hybrids obtained (*S. melongena* EG203, *S. incanum* UPV1, *S. insanum* UPV2, and *S. anguivi* UPV3), and the four interspecific hybrids obtained at four weeks after inoculation with *Ralstonia solanacearum* strains Pss97 or Pss2016. The number of plants used for each genotype and strain is indicated (N).

Genotype	Strain Pss97 ^a^				Strain Pss2016 ^a^			
	N	W%	DI%	Reaction	N	W%	DI%	Reaction
*S. melongena* EG048	9	100.0	100.0	S	9	100.0	77.8	S
*S. melongena* EG203	9	11.1	8.9	R	9	33.3	20.0	R
*S. incanum* UPV1	24	100.0	100.0	S	24	66.7	23.3	R
*S. insanum* UPV2	24	87.5	85.0	S	24	100.0	96.7	S
*S. anguivi* UPV3	24	100.0	100.0	S	24	95.8	45.8	MR
*S. melongena* EG203 × *S. incanum* UPV1	12	83.3	56.7	S	16	94.4	56.4	S
*S. incanum* × *S. melongena* EG203	19	90.5	67.6	S	19	95.2	58.1	S
*S. insanum* UPV2 × *S. melongena* EG203	12	100.0	98.0	S	12	100.0	95.0	S
*S. anguivi* UPV3 × *S. melongena* EG203	12	100.0	90.0	S	12	100.0	85.0	S

^a^ W% and DI% indicate the means of three replications of wilt percentage and disease index, respectively, at the fourth week after inoculation. The reaction category was made according to the disease index (DI) at the fourth week after inoculation. R = resistant (0–30%), MR = moderately resistant (>30–40%), MS = moderately susceptible (>40–50%), S = susceptible (>51%).

**Table 5:** Cultivated eggplant and wild relatives were used for interspecific hybridization experiments.

Species	Accession Code	Country of Origin	Gene Pool
*Solanum melongena*	EG203	India	Cultivated
*S. incanum*	UPV1	Israel	Secondary
*S. insanum*	UPV2	Sri Lanka	Primary
*S. anguivi*	UPV3	Ivory Coast	Secondary
*S. sisymbriifolium*	UPV4	Unknown	Tertiary

**Table 6:** DNA sequence of primers, PCR conditions, and characteristics of the Simple Sequence Repeat (SSR) markers used for hybridity confirmation for interspecific hybrids developed between cultivated eggplant and wild relatives.

Marker	Forward Primer	Reverse Primer	Primer Melting Temperature (°C)	Band Size (bp)	Repeat Motif
smSSR01	GTGACTACGGTTTCACTGGT	GATGACGACGACGATAATAGA	50	310	(ATT)_21_
EPSSR04	AATGAGTCAGAAACCACGCC	CGTTTAACCTTTGGCTCGAA	55	147	(CA)_10.5_
EPSSR133	AGTGGTAACGTCTGCTTACATTT	AGTTTGAATTCCATGGCTCG	55	230	(TGT)_9_
